# Effectiveness of a Step Counter Smartband and Midwife Counseling Intervention on Gestational Weight Gain and Physical Activity in Pregnant Women With Obesity (Pas and Pes Study): Randomized Controlled Trial

**DOI:** 10.2196/28886

**Published:** 2022-02-15

**Authors:** Elena Gonzalez-Plaza, Jordi Bellart, Ángela Arranz, Leila Luján-Barroso, Esther Crespo Mirasol, Gloria Seguranyes

**Affiliations:** 1 Maternal-Fetal Medicine Department at BCNatal Clinic Hospital of Barcelona Barcelona Spain; 2 Department of Nursing, Public, Mental and Maternity and Child Health School of Nursing, Faculty of Medicine and Health Science University of Barcelona Barcelona Spain; 3 Department of Medicine University of Barcelona Barcelona Spain; 4 Unit of Nutrition and Cancer, Cancer Epidemiology Research Programme Bellvitge Biomedical Research Institute Catalan Institute of Oncology Barcelona Spain; 5 Research Group on Sexual and Reproductive Health Care (GRASSIR) Barcelona Spain

**Keywords:** obesity, maternal, pregnancy, mHealth, mobile apps, telemedicine, telenursing, physical activity, gestational weight gain, lifestyle, mobile phone

## Abstract

**Background:**

Women who are pregnant and have obesity and excessive gestational weight gain (GWG) present a higher risk of maternal and perinatal complications. The use of mobile apps and a wristband during pregnancy may contribute to promoting healthy lifestyles and, thus, improving maternal and neonatal health.

**Objective:**

This study aims to evaluate the effectiveness of a complex digital health intervention, using a smartband and app with midwife counseling, on GWG and physical activity (PA) in women who are pregnant and have obesity and analyze its impact on maternal and perinatal outcomes. In addition, we aim to study the frequency of use, usability, and satisfaction with the mobile apps used by the women in the intervention group.

**Methods:**

A parallel, 2-arm, randomized controlled trial was conducted. A total of 150 women who were pregnant and had obesity were included. The intervention group received a complex combined digital intervention. The intervention was delivered with a smartband (Mi Band 2) linked to the app Mi Fit to measure PA and the Hangouts app with the midwife to provide personal health information. The control group received usual care. The validated Spanish versions of the International Physical Activity Questionnaire–Short Form and the System Usability Scale were used. Satisfaction was measured on a 1- to 5-point Likert scale.

**Results:**

We analyzed 120 women, of whom 30 (25%) were withdrawn because of the COVID-19 pandemic. The median GWG in the intervention group was 7.0 (IQR 4-11) kg versus 9.3 (IQR 5.9-13.3) kg in the control group (*P=*.04). The adjusted mean GWG per week was 0.5 (95% CI 0.4-0.6) kg per week in the control group and 0.3 (95% CI 0.3-0.4) kg per week in the intervention group (*df*=0.1, 95% CI −0.2 to 0.03; *P*=.008). During the 35 and 37 gestational weeks, women in the intervention group had higher mean PA than women in the control group (1980 metabolic equivalents of tasks–minutes per week vs 1386 metabolic equivalents of tasks–minutes per week, respectively; *P=*.01). No differences were observed between the study groups in the incidence of maternal and perinatal outcomes. In the intervention group, 61% (36/59) of the women who were pregnant used the smartband daily, and 75% (44/59) evaluated the usability of the Mi Fit app as excellent. All women in the intervention group used the Hangouts app at least once a week. The mean of the satisfaction scale with the health counseling app and midwife support was 4.8/5 (SD 0.6) points.

**Conclusions:**

The use of a complex mobile health intervention was associated with adequate GWG, which was lower in the intervention group than in the control group. In addition, we observed that the intervention group had increases in PA. No differences were observed in maternal perinatal complications.

**Trial Registration:**

ClinicalTrials.gov NCT03706872; https://www.clinicaltrials.gov/ct2/show/NCT03706872

## Introduction

### Background

Obesity during pregnancy is an increasingly prevalent public health problem in society. Prepregnancy obesity in Europe was estimated to be 7.8% to 25.6% [1]. It involves a greater risk of maternal and neonatal complications such as gestational diabetes, preeclampsia a pregnancy-induced hypertension disorders, a high rate of cesarean sections, fetal prematurity, macrosomy, and newborns large for gestational age (LGA) [2,3]. Women with excessive gestational weight gain (GWG) have a higher probability of presenting complications [4], which increases according to the class of obesity [5]. The *Institute of Medicine* (IOM) recommends a weight gain during pregnancy between 5 kg and 9 kg in women with obesity to minimize complications [6].

### Prior Work

Interventions promoting physical activity and healthy food habits in women who are pregnant have been effective in limiting GWG and have been associated with a reduction in diabetes, cesarean sections, and macrosomy [7,8]. However, these interventions have not demonstrated a reduction in maternal and neonatal complications in women who are pregnant and overweight and have prepregnancy obesity [9]. Furthermore, several studies in women who are pregnant and have obesity have described low adherence to the intervention [10]; thus, promoting healthy lifestyles using new information and communication technologies (ICTs) may be useful for health care professionals and are accessible to a larger population [11].

ICTs enhance self-control, self-evaluation, self-reinforcement, and personalized feedback through monitoring devices. Thus, ICT interventions based on social cognitive theory could be useful in promoting healthy habits in women who are pregnant [12].

Mobile health allows access to and receipt of health information, which may contribute to the promotion of healthy lifestyles and improvement of maternal and neonatal health [13].

In Spain, 97.7% of Spanish women aged between 16 and 54 years seek information on the internet on topics related to health, and 99.9% use their mobile telephone to do so [14]. According to a meta-analysis by Lau et al [15], 70% of women who are pregnant and overweight or have obesity consulted a webpage or used a mobile app to obtain information on adequate GWG. Furthermore, this meta-analysis reported a limiting GWG and self-reported increase in moderate physical activity during the postpartum period in the electronic-based lifestyle intervention group in women who are overweight or have obesity.

In addition, in the past years, wearable devices such as wristbands have emerged. In 2019, a total of 62.9 million wristband units were sold, with a trend toward a rise in sales being foreseen [16]. Wristbands help monitor different aspects of health habits, including monitoring of physical activity. One of the most commonly used wristbands is the smartband. These devices incorporate gamification functions; for example, challenges and prizes that increase commitment to digital health interventions [13].

There is emerging evidence that pedometer interventions may be successful in increasing activity levels in women who are pregnant and have obesity.

A recent intervention study on the feasibility of using a pedometer in women who are pregnant and have prepregnancy obesity reported promising results in GWG and increased physical activity [17]. Despite the growing number of women who are pregnant consulting the internet or using smartbands during pregnancy, few studies have analyzed the impact of their use in women who are pregnant and have prepregnancy obesity [15].

### Objective

The aim of the study is to evaluate the effectiveness of a complex digital health intervention, using a smartband and app with midwife counseling, on GWG and physical activity in women who are pregnant and have obesity. The secondary objectives are to assess the impact of these interventions on maternal and perinatal outcomes and identify the frequency of use, usability, and satisfaction with the mobile apps used by the women in the intervention group.

## Methods

### Study Design

This randomized parallel controlled trial (Pas and Pes; from Catalan language, *weight* and *step*) with 2 arms in a 1:1 (intervention and control group) ratio was conducted at the maternal–fetal department of the Hospital Clinic of Barcelona from June 2018 to October 2020. The trial was registered on the Clinical Trial Register of the National Library of Medicine of the United States (NCT03706872).

### Recruitment

Eligible participants were women who were pregnant and had prepregnancy obesity (BMI ≥30 kg/m^2^ based on World Health Organization classification [18]) who attended hospital obstetric clinics during prenatal care.

Women who were pregnant and had prepregnancy BMI ≥30 kg/m^2^ at 12 to 18 weeks of pregnancy, singleton pregnancy, aged ≥18 years, users of an Android smartphone or iPhone (iOS) with an internet connection, and who agreed to participate were included in the study.

The exclusion criteria were women who were pregnant who had already used an app for monitoring physical activity and weight. Women with a previous diagnosis of psychiatric disorders, endocrine–metabolic disorders, or chronic hypertension; pregnant women with a contraindication for performing exercise or mobility problems that do not allow moderate walking; and women with language difficulties in understanding Spanish were also excluded.

All women were recruited by midwives.

Women who were pregnant and attending hospital obstetric clinics who fulfilled the inclusion criteria were consecutively included in the study.

All participants provided written informed consent before being fully enrolled in the study.

### Sample Size

The sample size calculation was based on the variable of weight gain to detect a difference ≥3.4 (SD 7.1) kg [19]. An α risk of .05 and a β risk of .2 were accepted in the bilateral contrast. It was calculated that 81 women were needed in the intervention group, and 81 women were needed in the control group. A loss to follow-up of 20% was estimated.

### Randomization

Randomization was computer based. Two random number lists were created by the University of Barcelona, and opaque numbered envelopes were prepared to mask the group assignment.

After the study participant had been informed about the study, and they accepted and signed the informed consent, the midwife opened the opaque and sealed envelope, and the woman who was pregnant was assigned to either the intervention or control group.

### Intervention

#### Usual Prenatal Care in the Control and Intervention Groups

**Figure 1:**
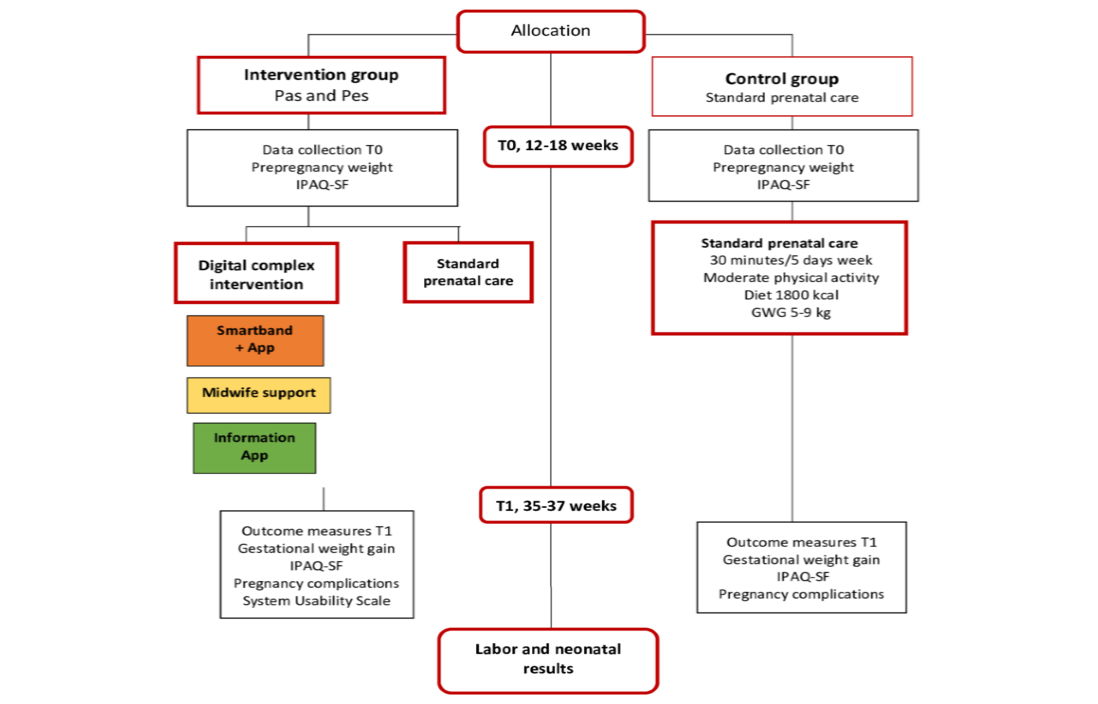
Flowchart of the Pas and Pes study. GWG: gestational weight gain; IPAQ-SF: International Physical Activity Questionnaire–Short Form; T0: time 0; T1: time 1.

All the study participants received the standard prenatal care by midwives and obstetricians according to the *Pregnancy Monitoring Protocol* in Catalonia [20], which also includes health education in relation to physical activity GWG and food habits (Figure 1).

#### Characteristics of the Intervention in the Intervention Group

A complex digital intervention, based on social cognitive theory [12], was performed as a behavior-changing strategy of self-control, self-efficacy, and improvement of outcome expectations and to address barriers to the use of a smartband and an app for receiving information and support from a midwife.

#### Smartband (Mi Band 2) and Mi Fit App

After participants were assigned to the intervention group, the midwife gave the participants a smartband (Mi Band 2) and explained that it should be worn during the day. Women who were pregnant were recommended to take 10,000 steps a day, equivalent to 30 minutes per day of moderate physical activity [21], over the week (≥5 days) according to the recommendations of the American College of Obstetricians and Gynecologists [22]. The smartband was linked to the Mi Fit app, which was free and available for Android and iOS systems. The midwife instructed the intervention group’s participants on how to set up the step and weight goals through notifications of goals and activated alerts in the Mi Fit app. The smartband would vibrate during prolonged periods of inactivity or send prizes when goals were achieved. Women verified objective fulfillment by alerts and notifications from the Mi Fit app and the smartband (Mi Band 2).

#### Hangouts App

The app for receiving health counseling and support from a midwife was Hangouts (Google LLC).

If necessary, the midwife instructed the women on how to download the app, which was free and available for Android and iOS systems (Hangouts), so that pregnant women could receive personalized information through SMS text messages or videos sent by the research team twice a week. One message corresponded to information regarding the physiological changes in the mother and fetus, and another was related to healthy eating habits, weight gain, physical activity, and information related to pregnancy, labor, and postpartum. The messages were personalized according to the gestational week and could contain videos (Multimedia Appendix 1). The source of information of the sent messages was extracted from a specialized webpage and the Inatal app. This specialized webpage is a social web designed by gynecologists and midwives from the Hospital Clinic, Barcelona. Permission was obtained for using its content. Video links for promoting physical activity and healthy eating habits were used from the webpage of the *Health Department of Catalonia*. Finally, we used videos and informative material from the *Catalan Midwives Association* available on their website. The women were to use the Hangouts app at least once a week.

In addition, the midwife asked the women who were pregnant about their current weight and motivated or reinforced their progress monthly (one by one woman) through the Hangouts app. Furthermore, women who were pregnant could ask questions to the midwife that were solved with an immediate response (<1 hour). No information regarding the data or results in the clinical history of the woman was delivered through Hangouts app (Figure 1).

#### Characteristic of the Intervention in Control and Intervention Group

Women who were pregnant in the control group received oral information and written support material. With respect to physical activity, it was recommended to perform 30 min/day of moderate physical activity over the week (≥5 days) according to the recommendations of the American College of Obstetricians and Gynecologists [22]. Midwives gave instructions to gradually achieve the goal in those who were inactive or sedentary. Furthermore, midwives recommended a GWG between 5 kg and 9 kg to women who were pregnant, according to the IOM [6], and a balanced (Mediterranean) diet of 1800 kcal.

### Outcomes and Data Collection

#### Main Outcome Variables

The main outcome variables were GWG and total physical activity. GWG was obtained by the difference between the weight of the woman who was pregnant between weeks 35 and 37 of pregnancy or time 1 (T1) and self-reported prepregnancy weight at the time of recruitment or time 0 (T0) and the mean GWG adjusted by the week of pregnancy in the study. The midwife weighed the dressed and shoeless woman who was pregnant in the midwife consultation using a Seca 704 scale. GWG was categorized according to the IOM recommendations as below (<5 kg), within (5-9 kg), and above the guidelines (>9 kg) [6].

Total physical activity was calculated using the global score of the *International Physical Activity Questionnaire–Short Form* [23,24], which participants self-reported at T0 and T1. The volume of physical activity was determined using metabolic equivalent of task (MET) units [25] and was calculated as METs-minutes per week. In addition, information on the types of physical activity was obtained: vigorous, moderate, and walking. Total physical activity was obtained by category (category 1 or low [≤600 METs-minutes per week], category 2 or moderate [600-3000 METs-minutes per week], and category 3 or high [>3000 METs-minutes per week]) and sitting time (minutes per week).

#### Secondary Outcome Variables

Secondary outcomes variables were as follows:

- The incidence of maternal complications was a miscarriage at 22 weeks, gestational diabetes according to the diagnostic criteria of the International Association of Diabetes and Pregnancy Study Groups [26], preeclampsia or pregnancy-induced hypertensive disorder [27], and prematurity. The variable pregnancy composite morbidity (yes or no) was created, where it was coded as yes if the woman who was pregnant presented at least one of the four adverse results found during pregnancy.
- Incidence of birth induction, type of delivery, and unplanned cesarean section.
- Incidence of perinatal complications was macrosomy (weight >4000 g), low birth weight (weight <2500 g), small for gestational age (percentile <10) and LGA (percentile >90) adjusted for newborn sex, postterm newborn, and neonatal death. The variable perinatal composite morbidity (yes or no) was created, which included the 7 adverse perinatal results mentioned above together with the variable of prematurity. Admissions to the neonatal intensive care unit were monitored.

Although the same woman or newborn might have had ≥1 adverse outcome during the process, the pregnancy composite morbidity and perinatal composite morbidity variables only counted as one event. Data on pregnancy complications were obtained by the midwives at T1. Data on delivery and newborns were retrospectively obtained by the research team through electronic clinical history.

Secondary specific outcome measures for the intervention group were frequency of smartband use and grade of usability of the Mi Fit app according to the total score and by categories (excellent, good, poor, and awful) of the System Usability Scale [28]. The satisfaction of women who received health counseling and midwife support through the app was evaluated with 6 questions answered using a 1- to 5-point Likert scale in which 1=not at all satisfied and 5=very satisfied. Self-reported questionnaires were answered anonymously by the women who were pregnant.

### Statistical Analysis

The analyses were performed on an intention-to-treat basis according to the treatment group allocated at randomization.

Descriptive data were presented as numbers and percentages, means and SDs, and medians and IQR. Bivariate analysis was performed between sociodemographic variables and prepregnancy BMI. For comparison of categorical variables, the nonparametric test of chi-square or Fisher exact (in case of small sample size of compared groups and expected frequency <5) and McNemar test were used. To compare quantitative variables, parametric Student *t* test (2-tailed) and nonparametric Mann–Whitney *U* or Wilcoxon tests were performed, depending on the normality distribution of compared groups.

Multinomial logistic regression was used to analyze the association between total physical activity (low, moderate, and high) at the end of the study, age and BMI at recruitment, previous births (yes or no), and test group (control and intervention). Adjusted odds ratios and 95% CIs were calculated for each model.

To evaluate the effect of the intervention on GWG per week (kg per week) of the participants at the end of the study, a linear regression model was used, which was adjusted for age and BMI at recruitment, previous births (yes or no), and total physical activity (low, moderate, and high) at the end of the study. The adjusted GWG per week was derived from this model.

All statistical tests were 2-sided and evaluated at an α level of .05. Analyses were performed using SPSS (version 25) and SAS (version 9.4).

### Ethical Aspects

The study was approved by the ethics and clinical research committee of the Hospital Clinic of Barcelona (code HCB2017-0756). The anonymity and confidentiality of the data were always preserved in accordance with the Spanish Organic Law 3/2018, of December 5, on the Protection of Personal Data and guarantee of digital rights. Informed consent was obtained from all the participants.

## Results

### Participants

**Figure 2:**
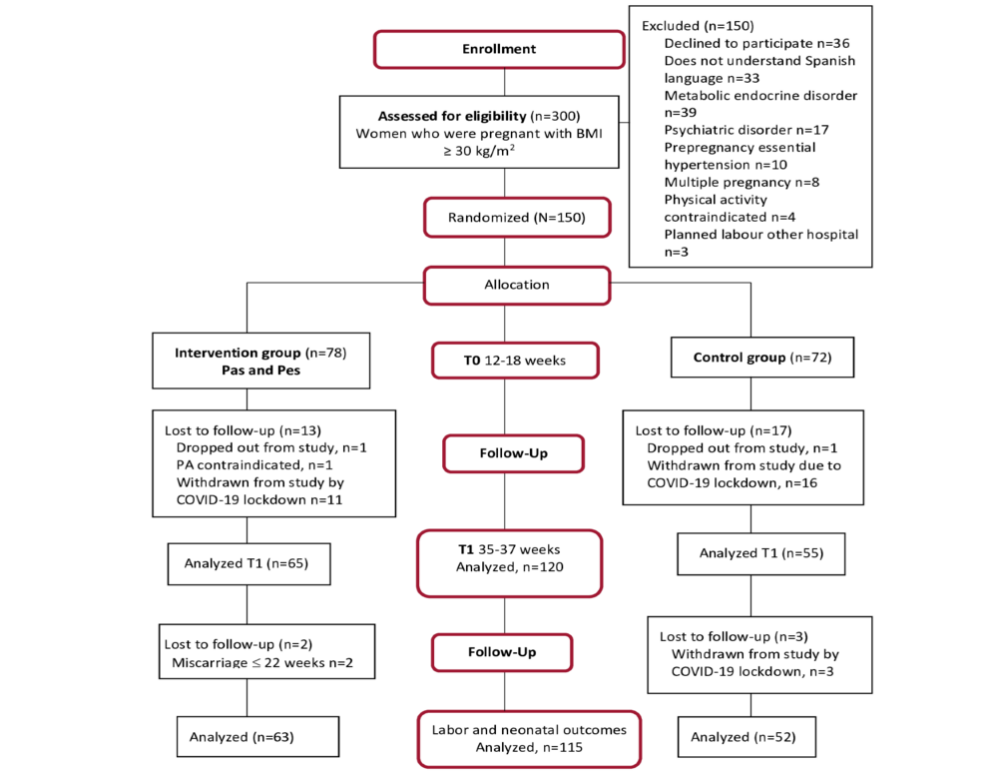
CONSORT (Consolidated Standards of Reporting Trials) flowchart of participants in the Pas and Pes study. PA: physical activity; T0: time 0; T1: time 1.

Figure 2 shows the flow diagram of the recruitment of study participants according to the recommendations of the CONSORT (Consolidated Standards of Reporting Trials) statement. Of the 300 women evaluated for recruitment, 150 (50%) fulfilling the inclusion criteria were randomized: 52% (78/150) in the intervention group and 48% (72/150) in the control group.

The COVID-19 pandemic in Spain led to strict home confinement, which interfered with the physical activity of women. As prenatal care was delivered only by telematic means on April 1, 2020, up to 20% (30/150) of women who had not reached 35 weeks of pregnancy were withdrawn from the study. At T1, of the 150 women, 120 (80%) were analyzed, and variables related to delivery and the neonates of 115 (76.7%) women were analyzed (Figure 2).

The baseline characteristics of the study participants are shown in Table 1. There were no statistically significant differences in age, country of origin, number of previous births, or prepregnancy BMI between the 2 groups. The mean follow-up was 21.5 (SD 3.2) weeks in the intervention group and 21.1 (SD 2.4) weeks in the control group (*P*=.48).

**Table 1:** Baseline demographics and clinical characteristics by treatment group (N=150).

Variables			Intervention group (n=78)		Control group (n=72)		*P* value
Age (years), mean (SD)			32.4 (5.4)		33.4 (4.7)		.36^a^
**Country of origin, n (%)**							.48^b^
	Spanish	40 (51)		41 (57)			
	Foreign	38 (49)		31 (43)			
**Educational level, n (%)**							.83^b^
	Primary	8 (10)		7 (10)			
	Secondary	37 (47)		31 (43)			
	Higher	33 (42)		34 (47)			
**Employed, n (%)**							.82^b^
	Yes	65 (83)		59 (82)			
	No	13 (17)		13 (18)			
**Cohabiting partner, n (%)**							.09^b^
	Yes	66 (85)		53 (74)			
	No	12 (15)		19 (26)			
**Prepregnancy weight (kg)**							.34^a^
	Values, mean (SD)	86.1 (10.4)		84.3 (9.9)			
	Values, median (IQR)	84 (79.7-92.3)		84 (77-90)			
**Prepregnancy BMI (kg/m^2^)**							.06^a^
	Values, mean (SD)	33.1 (2.9)		32.7 (3.3)			
	Values, median (IQR)	32.6 (31.1-34.2)		31.3 (30.4-33.6)			
**Obesity class, n (%)**							.55^b^
	Class I (30-34.9 kg/m^2^)	63 (81)		61 (85)			
	Class II (35-39.9 kg/m^2^)	13 (17)		8 (11)			
	Class III (≥40 kg/m^2^)	2 (2)		3 (4)			
**Previous births, n (%)**							.06^b^
	Yes	36 (46)		44 (61)			
	No	42 (54)		28 (39)			
**Smoking, n (%)**							.29^b^
	Yes	8 (10)		4 (6)			
	No	70 (90)		68 (94)			

^a^Mann–Whitney *U* test.

^b^Chi-square test.

### Main Outcomes

#### GWG Outcome

The intervention group median of GWG 7.0 (IQR 4-11) kg was statistically significantly lower than the control group median of 9.3 (IQR 5.9-13.3 kg; *P=*.04). At T1, the median GWG per week was 0.3 in the intervention group versus 0.4 in the control group (*P*=.01).

An inverse association was observed between the GWG (kg per week) at the end of the study in the intervention group compared with the control group (β=−.1, 95% CI −0.2 to −0.03) at the same levels of age, BMI at recruitment, physical activity, and previous births (Multimedia Appendix 2). Derived from the model, we obtained an adjusted mean weight gain per week that was 0.5 (95% CI 0.4-0.6) kg per week for the control group and 0.3 (95% CI 0.3-0.4) kg per week for the intervention group; (*df*=0.1, 95% CI −0.2 to 0.03; *P*=.008; Table 2).

**Table 2:** Gestational weight gain by study group (N=113)^a^.

Gestational weight gain		Intervention group (n=60)	Control group (n=53)	Mean difference (95% CI)		β		*P* value	
**Continuous (kg)**									
	Values, mean (SD)	7.6 (5.5)	10.1 (6.4)	2.5 (0.2 to 4.7)		N/A^b^		.02^c^	
	Values, median (IQR)	7.0 (4.0 to 11.0)	9.3 (5.9 to 13.3)	N/A		N/A		.04^d^	
**Weekly weight gain (kg)**									
	Values, mean (SD)	0.3 (0.3)	0.4 (0.3)	0.1 (0.03 to 0.2)		N/A		.01^e^	
	Values, median (IQR)	0.3 (0.2 to 0.5)	0.4 (0.3 to 0.6)	N/A		N/A		.01^d^	
	Adjusted mean	0.3	0.5	0.1 (−0.2 to −0.03)		−.1		.008^e^	
**Categorical based on IOM^f^ guidelines, n (%)**					N/A		N/A		.08^g^
	Below guidelines	18 (30)	9 (17)						
	Within guidelines	21 (35)	15 (28)						
	Above guidelines	21 (35)	29 (55)						

^a^N=113; missing data of 2 miscarriages and 5 premature deliveries at ≤35 weeks.

^b^N/A: not applicable.

^c^Student *t* test (2-tailed).

^d^Mann–Whitney *U* test.

^e^*P* value adjusted for age (years), BMI at time 0, and previous births (yes or no).

^f^IOM: Institute of Medicine*.*

^g^Chi-square test.

The proportion of women with adequate GWG according to IOM recommendations was 35% (21/60) in the intervention group versus 28% (15/53) in the control group; GWG below guidelines was 30% (18/60) in the intervention group versus 17% (9/53) in the control group; and GWG above guidelines was 35% (21/60) in the intervention group versus 55% (29/53) in the control group (*P=*.08; Table 2).

#### Physical Activity

**Figure 3:**
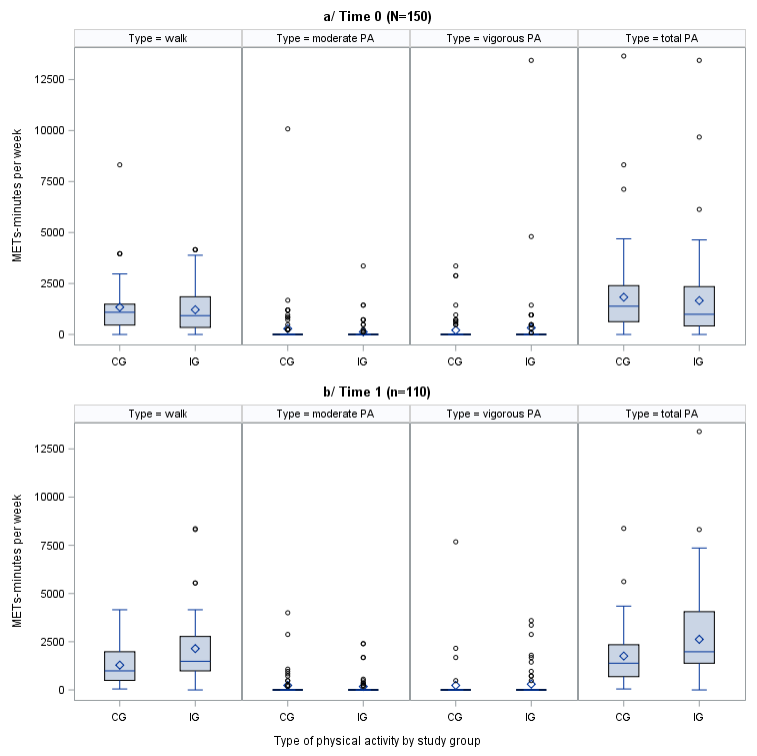
Physical activity by study group at time 0 and time 1 (time 0=12-18 weeks; time 1=35-37 weeks). CG: control group; IG: intervention group; MET: metabolic equivalent of task; PA: physical activity.

Regarding total physical activity, in intragroup comparison, women in the intervention group performed greater total physical activity at T1 than at T0 (1980 vs 990 METs-minutes per week; *P*=.001), whereas women in the control group did not modify their METs-minutes per week at T1 compared with T0 (*P*=.69). When we compared the 2 groups at T1 (intervention group vs control group), women in the intervention group had higher mean total physical activity than women in the control group (1980 METs-minutes per week vs 1386 METs-minutes per week; *P*=.01; Figure 3 and Table 3). Regarding sitting time, women in the intervention group obtained a lower mean of 1260 minutes per week than 2100 minutes per week in the control group (*P=*.02; Table 3).

**Table 3:** Intragroup physical activity outcomes by period time 0 (T0) and time 1 (T1) and physical activity outcomes in T1 by study group (N=110)^a^.

Physical activity		Intervention group (n=59)		Control group (n=51)		*P* value
		T0 (12-18 weeks)	T1 (35-37 weeks)	T0 (12-18 weeks)	T1 (35-37 weeks)	
**Total**						
	Values (METs^b^-minutes per week), median (IQR)	990 (396-2376)	1980 (1386-4060)	1386 (495-2685)	1386 (693-2346)	.01^c^
	Values (METs-minutes per week), minimum-maximum	0-13,400	0-13,400	0-8316	50-8373	.01^c^
	*P* value^d^ (intragroup)	N/A^e^	<.001	N/A	.69	N/A
**Vigorous**						
	Values (METs-minutes per week), median (IQR)	0 (0-0)	0 (0-0)	0 (0-0)	0 (0-0)	.67^c^
	Values (METs-minutes per week), minimum-maximum	0-13,440	0-3600	0-3360	0-7680	.67^c^
	0 METS-minute per week, n (%)	47 (80)	49 (83)	42 (82)	47 (92)	N/A
	*P* value^d^ (intragroup)	N/A	.83	N/A	.23	N/A
**Moderate**						
	Values (METs-minutes per week), median (IQR)	0 (0-0)	0 (0-0)	0 (0-0)	0 (0-0)	.16^c^
	Values (METs-minutes per week), minimum-maximum	0-3360	0-2400	0-1680	0-4000	.16^c^
	0 METS-minutes per week, n (%)	48 (81)	40 (78)	39 (76)	48 (81)	N/A
	*P* value^d^ (intragroup)	N/A	.93	N/A	.97	N/A
**Walking**						
	Values (METs-minutes per week), median (IQR)	693 (330-1782)	1485 (990-2772)	693 (346.5-1980)	990 (495-1980)	.003^c^
	Values (METs-minutes per week), minimum-maximum	0-4158	0-8360	0-8316	50-4158	.003^c^
	0 METS-minutes per week, n (%)	4 (7)	1 (2)	1 (2)	0 (0)	N/A
	*P* value^d^ (intragroup)	N/A	<.001	N/A	.55	N/A
**Physical activity by category**						
	Category I: low, n (%)	22 (37)	6 (10)	15 (29)	12 (3)	.10^f^
	Category II: moderate, n (%)	30 (51)	36 (61)	29 (57)	30 (59)	.10^f^
	Category III: high, n (%)	7 (12)	17 (29)	7 (14)	9 (18)	.10^f^
	*P* value^g^ (intragroup)	N/A	<.001	N/A	.83	N/A
**Sitting time (minutes per week)^h^**						
	Values (METs-minutes per week), median (IQR)	1680 (840-2940)	1260 (420-2100)	1680 (840-2940)	2100 (1260-2520)	.02^c^
	Values (METs-minutes per week), minimum-maximum	0-5880	55-5460	20-5040	55-7560	.02^c^
	*P* value^g^ (intragroup)	N/A	.16	N/A	.81	N/A

^a^N=110; missing data of 2 miscarriages, 5 premature deliveries at ≤35 weeks, and 3 non–International Physical Activity Questionnaire–Short Form data.

^b^MET: metabolic equivalent of task.

^c^Wilcoxon test.

^d^Mann–Whitney *U* test of the period time 1 data intervention group versus time 1 control group.

^e^N/A: not applicable.

^f^Chi-square test.

^g^McNemar test of the period time 1 data intervention group versus time 1 control group.

^h^In the intervention group, n=52 at time 0 and n=54 at time 1 and in the control group, n=47 at time 0 and n=45 at time 0.

Multimedia Appendix 3 shows results of the multinomial logistic regression for categorical physical activity. The probability of high versus low physical activity, with the other variables in the model remaining constant, was 3.9-fold higher in the intervention group (95% CI 1.1-14.3) than in the control group.

### Secondary Outcomes

#### Maternal and Perinatal Complications During Pregnancy and Delivery

Pregnancy, labor, and perinatal complications by study group are detailed in Table 4.

**Table 4:** Pregnancy, labor, and perinatal complications by study group (N=120).

Complications				Intervention group (n=65)	Control group (n=55)	*P* value	
**Gestational complications, n (%)**							
	Composite pregnancy morbidity			22 (34)	20 (36)	.77^a^	
	Miscarriage ≤22 weeks			2 (3)	0 (0)	.49^b^	
	Gestational diabetes^c^			10 (15)	12 (22)	.36^a^	
	Preeclampsia or gestational hypertension^c^			6 (9)	9 (16)	.23^a^	
	Preterm labor ≤37 weeks^d^			7 (11)	5 (9)	.17^a^	
**Labor complications, n (%)^e,f^**							
	**Type of labor onset**						.15^a^
		Spontaneous		23 (38)	22 (44)		
		Induction		26 (43)	25 (50)		
		Planned cesarean		11 (18)	3 (6)		
	**Type of labor**						
		Vaginal		37 (62)	39 (78)	.06^a^	
		**Cesarean**		23 (38)	11 (22)	.06^a^	
			Planned	11 (48)	3 (27)	.29^b^	
			Unplanned	12 (52)	8 (73)	.29^b^	
**Perinatal complications, n (%)^d,g^**							
	Composite perinatal morbidity			24 (38)	24 (46)	.38^a^	
	Birthweight ≥4000 g			4 (6)	6 (12)	.34^b^	
	Birthweight ≤2500 g			5 (8)	2 (4)	.45^b^	
	**Large for gestational age centiles**						.87^b^
		≤5th		6 (10)	3 (6)		
		5-10th		2 (3)	1 (2)		
		10-90th		43 (68)	37 (71)		
		≥90th		12 (19)	11 (21)		
	Postterm			7 (11)	8 (15)	.49^a^	
	**Perinatal death**			1 (2)	2 (4)	.58^b^	
		Early neonatal death		1 (100)	1 (50)		
		Antepartum stillbirth		0 (0)	1 (50)		
	Admission to NICU^h,i^			4 (6)	5 (10)	.72^b^	

^a^Chi-square test.

^b^Fisher exact test.

^c^N=118; missing data for 2 miscarriages.

^d^N=115; missing data for 2 miscarriages and 3 COVID-19 lockdowns in delivery.

^e^N=110, missing data of 2 miscarriages, 5 premature deliveries at ≤35 weeks, and 3 COVID-19 lockdowns in delivery.

^f^n=60 for the intervention group and n=50 for the control group.

^g^n=63 for the intervention group and n=52 for the control group.

^h^NICU: neonatal intensive care unit.

^i^N=114; missing data for 2 miscarriages, 3 COVID-19 lockdowns in delivery, and 1 antepartum stillbirth.

#### Frequency of Using Mi Band 2 and Hangouts App, Grade of Usability Mi Fit App, and Grade of Satisfaction in Women in the Intervention Group (Hangouts) App

Information was obtained from 91% (59/65) of the women in the intervention group at T1. None of the women showed adverse effects with the use of the smartband (Mi Band 2). The smartband was used daily by 61% (36/59) of the women. The mean System Usability Scale score of the app linked to the smartband (Mi Fit) was 89.7 (SD 14.9) points, and 75% (44/59) evaluated its use as excellent. All 59 women reported having consulted the information provided in the app (Hangouts). All these women used the Hangouts app at least once a week, and they received midwives’ feedback once a month and every time they formulated questions. The mean grade of overall satisfaction with receiving messages related to pregnancy and health counseling and midwife support through the app was 4.8/5 (SD 0.6) points (Table 5).

**Table 5:** Usability score of the Mi Fit app, frequency of Mi Band 2 and Hangouts app use, and grade of satisfaction with Hangouts app in the intervention group (N=59)^a^.

Measures			Values
Mi Fit app: usability score (SUS^b^), mean (SD)			89.7 (14.9)
**Mi Fit app: usability score (SUS)—categorical, n (%)**			
	Excellent (≥80.3)	44 (75)	
	Good (68 to 80.3)	8 (14)	
	Poor (51-67)	6 (10)	
	Awful (≤51)	1 (2)	
**Frequency of smartband use (Mi Band 2), n (%)**			
	Daily	36 (61)	
	3-4 times per week	11 (19)	
	2 times per week	6 (10)	
	1 time per week	5 (10)	
	Never	0 (0)	
**Satisfaction with app information (Hangouts), mean (SD)**			
	Utility of pregnancy advice	4.6 (0.6)	
	Utility of healthy lifestyles advice	4.6 (0.6)	
**Satisfaction with midwife support by (Hangouts) app, mean (SD)**			
	Midwife accessibility	4.7 (0.7)	
	Ease of use of the chat	4.7 (0.8)	
	Be able to take advice without having to scroll	4.7 (0.6)	
Global satisfaction, mean (SD)			4.8 (0.6)

^a^The grade of satisfaction was analyzed with a Likert scale in which the minimum grade of satisfaction was 1 point and maximum 5 points; N=59; missing data of 2 miscarriages at 22 weeks, 3 premature deliveries at 35 weeks, and 1 no data of System Usability Scale and of the satisfaction questionnaire scale.

^b^SUS: System Usability Scale.

## Discussion

### Principal Findings

Our findings show that the use of a complex digital intervention was associated with lower GWG and an increase in physical activity during pregnancy in women who were pregnant and had obesity. No differences in the incidence of maternal and perinatal complications between the 2 study groups were found. All women in the intervention group used the smartband and health counseling app at least once a week. In addition, the usability of the app linked to the smartband was evaluated as excellent, and the grade of overall satisfaction with the health counseling app and support by the midwife was very high.

### Relation to Prior Literature

Recent research has suggested that interventions promoting healthy lifestyles and self-control using social networks of mobile apps in women who are pregnant have a moderate or low effect on maternal weight control [11,13]. Moreover, interventions accompanied by the use of self-monitoring devices [11] or those combined with professional reinforcement [13] are more effective for weight management.

In relation to GWG, our findings showed a mean difference in weight gain of 2.5 kg between the 2 groups, being lower in the intervention group. This GWG was lower than that reported in previous randomized controlled trials (RCTs) performed in women who were pregnant and had prepregnancy obesity. The intervention in those studies was using new technologies independently or combined with professional reinforcement through the sending of SMS text messages [29], social networks [30], telephone reinforcement [31], or with pedometers and telephone calls [32-34].

Pollak et al [35], based on n=34 (22 women in the chat group and 12 women in the SMS text messaging group) women who were pregnant and had obesity, provided health counseling for the management of GWG through SMS text messaging and a chat with professionals and observed a difference of 2.7 kg, which was similar to the GWG observed in our study. However, a recent RCT that included n=30 (10 per group for control, app, and app-coach) women who were pregnant and had obesity achieved a difference of 5.3 kg between the women who were pregnant and used a smartwatch linked to an app and the women who were pregnant and underwent an in-person coaching intervention or the group that used a smartwatch [17].

Similar to other studies, we observed that the proportion of excessive GWG of the women who were pregnant in the intervention group was lower than that of the control group [30,33]. In addition, we found a higher proportion of women with GWG <5 kg according to IOM [31,33].

Regarding physical activity, we showed that women who were pregnant and used the smartband and the app were more active, similar to the studies of Renault et al [33] and Poston et al [32]. Furthermore, our study and the study by Simmons et al [31] observed that women in the intervention group also spent less time sitting than women in the control group. In addition, we found that women in the intervention group increased their physical activity at T1 compared with T0, which was derived from the increase in physical activity by walking, as described by Darvall et al [17]. We observed 4 women with vigorous or high moderate physical activity, 3 (75%) of whom were derived from occupational physical activity and low walking physical activity at T0. At T1, those women decreased their occupational physical activity (probably because of increased onset of the usual symptoms of pregnancy between 35 and 37 weeks) and increased their walking physical activity.

Our results are in line with those based on a systematic review by Hussain et al [36], where an intervention combining several technological resources, such as the smartband with a reinforcement app with information and support from a midwife, was associated with better results in weight gain and physical activity during pregnancy.

With respect to maternal and perinatal complications, no differences were observed between the 2 groups, as in the previously mentioned RCT, although there was a trend toward presenting a lower incidence of gestational complications. As in other studies, there was a lower incidence of gestational diabetes [33,37], preeclampsia [33,37], macrosomy [33] and LGA [29,32], postterm newborns, and lower admission to the neonatal intensive care unit [30] in the intervention group than in the control group. However, there was a higher incidence of prematurity [29,30], small for gestational age or restricted intrauterine growth [29,32], and low newborn birth weight, in contrast to what was described by Poston et al [32].

Contrary to other studies [30,32,33], we observed a greater proportion of cesarean sections in the intervention group, similar to the findings of Okesene-Gafa et al [29]. However, there was a lower incidence of unplanned cesarean sections in the intervention group than in the control group, probably because of the higher incidence of planned cesarean sections in the intervention group.

In our study, the frequency of the use of the smartband and the linked app was very high, as all women who were pregnant in the intervention group used them at least once a week, similar to the study by Baruth et al [38]. This finding contrasts with the low adherence reported in the UK Pregnancies Better Eating and Activity Trial [32,39] or the RCT of Ainscough et al [40] and Szmeja et al [41] in women who were pregnant and overweight and had obesity.

The usability of the Mi Fit app linked to the smartband in our study was evaluated as excellent, as in the RCT Fit4two [42]. We observed that satisfaction with the messages and midwife support through the app was very high, and the acceptability of the intervention agreed with other RCTs in women who were pregnant and overweight and had obesity, such as SMARTMOMS [43], txt4two [44], or studies with women who were pregnant and had any BMI, such as RCT Interact [45] and the RCTs of Choi et al [46] and Coughlin et al [47].

Taking all of this into account, the use of a smartband and providing information and the support by a midwife through an app could be recommended to promote physical activity and adequate weight gain in the prenatal control of women who are pregnant and have obesity. It would also be useful to provide evidence-based information and solve doubts from a distance as health professionals have described difficulties in the management of GWG in women who are pregnant and have obesity and a lack of time in the consultation [48]. In addition, telematics access provides the opportunity for professionals to gain access to a greater population, even to women who are pregnant and who less frequently attend health care centers. Finally, providing information through apps increases quality and safety in the care of women during pregnancy and contributes to reducing the heterogeneity of information regarding health and pregnancy that women who are pregnant see on the internet [49].

### Strengths and Limitations

The strengths of this study include the ability to evaluate the effectiveness of a complex digital intervention by the use of a wearable device and apps in a clinical study, taking into account the increasing use of these devices worldwide in women with prepregnancy obesity. Randomized assignment to the intervention reduced the probability of selection bias and ensured that the study groups were homogeneous. Furthermore, this study provides information related to the usability of the app linked to the smartband using a validated questionnaire widely used by the scientific community. Similarly, we describe information on the frequency of use and satisfaction with the app with which the women who were pregnant received information and could consult midwives regarding doubts.

The main limitation of this study is that the estimated sample size could not be achieved because of the COVID-19 pandemic. Approximately 20% (30/150) of women who were pregnant included in our study were confined at home during the first wave of the pandemic (from March 14, 2020) and had to be withdrawn from the analysis of the study as we considered that this could influence the results, as the power of the analysis reduced to 63%. Nonetheless, these women continued in the study, and the results obtained are pending publication. The reduced sample size may have contributed to the lack of statistically significant differences in the trend of presenting less gestational diabetes, preeclampsia, and macrosomy observed in the intervention group, and, in turn, the remaining observed findings could not demonstrate a size effect because of limited statistical power. However, multinominal models were performed to adjust the effect of the intervention on the weight gain variables and physical activity by categories, showing that GWG was lower in the intervention group than in the control group and that there was a 4-fold higher probability of the intervention group performing physical activity than the control group.

The data collected by the app linked to the smartband in relation to the number of steps or physical activity performed by the women who were pregnant in the intervention group was not monitored as the objective of the study was to compare the physical activity between the 2 groups at T0 and T1. We used the validated self-reported International Physical Activity Questionnaire–Short Form questionnaire, which may have induced a memory bias with underestimated or overestimated reporting by the women [50,51]. Nonetheless, this questionnaire has been used in multiple studies evaluating physical activity in the population [24] and in the pregnant population who are overweight and have obesity [32,52].

Regarding to the Hangouts app questions that pregnant women asked the midwife through the app, we have not performed qualitative analyses.

Finally, we did not measure body composition in pregnancy with fat percentage and total body water using bioelectrical impedance analysis. The clinical utility of body composition measurements in pregnancy is an ongoing future area of research.

### Conclusions

Our results suggest that the use of a complex mobile health intervention was associated with adequate GWG, which was lower in the intervention group than in the control group. In addition, we observed that the intervention group increased their physical walking activity, although it did not reduce maternal and perinatal complications compared with the control group. Furthermore, our findings provide some support for the effectiveness and safety of the use of a smartband and an app for providing health counseling and support from a midwife during pregnancy in women who are pregnant and have obesity, which could be applied to promote healthy lifestyles in prenatal control. The frequency of use; satisfaction with the smartband, health counseling app, and midwife support; and usability of the app linked to the smartband were satisfactorily evaluated.

The findings were obtained with a reduced sample size, and thus, the size effect should be interpreted with caution. Furthermore, clinical studies in larger samples of women who are pregnant and have prepregnancy obesity are necessary to evaluate the effectiveness and feasibility, if any, of the use of new technologies during pregnancy and their influence on maternal and perinatal health.

## Appendix

Multimedia Appendix 1 Summary of SMS text messages and videos delivered to women who were pregnant through the app in the intervention group.

Multimedia Appendix 2 Linear model of gestational weight gain variables with the adjustment variables of age, BMI, physical activity, and study group.

Multimedia Appendix 3 Multinomial model of physical activity variable with the adjustment variables of age, BMI, physical activity, and study group.

Multimedia Appendix 4 CONSORT-eHEALTH checklist (V 1.6.1).

## Abbreviations

CONSORT: Consolidated Standards of Reporting Trials
GWG: gestational weight gain
ICT: information and communication technology
IOM: Institute of Medicine
LGA: large for gestational age
MET: metabolic equivalent of task
RCT: randomized controlled trial
T0: time 0
T1: time 1
