# Supplementary material for: Effectiveness of a Step Counter Smartband and Midwife Counseling Intervention on Gestational Weight Gain and Physical Activity in Pregnant Women With Obesity (Pas and Pes Study): Randomized Controlled Trial
Source: JMIR Mhealth Uhealth. 2022 Feb 15;10(2):e28886. doi: 10.2196/28886 (PMC8889480; doi:10.2196/28886)
Supplement: Multimedia Appendix 1 [file mhealth_v10i2e28886_app1.docx]

Appendix 1. Summary of text messages and videos delivered to pregnant women through the App in the intervention group

| **Dietary advice** | |
| --- | --- |
|  | Healthy diet |
|  | Gestational weight gain |
|  | Diet advice for gestational symptoms |
|  | Food poisoning |
| **Physical activity advices** | |
|  | Physical activity advices |
|  | Pelvic floor exercises |
| **Pregnancy advice** | |
|  | Changes in pregnancy week by week |
|  | Changes in foetus week by week |
|  | Pregnancy symptoms |
|  | Trips |
|  | Occupational information |
|  | Sexual relations |
|  | Vaccines and medicines |
| **Labour advice** | |
| **Postpartum advice** | |
| **Breastfeeding advice** | |
| **COVID-19 information** | |
|  | COVID-19 information |
|  | COVID-19 and pregnancy, labour and breastfeeding |
