# Supplementary material for: Effectiveness of a Step Counter Smartband and Midwife Counseling Intervention on Gestational Weight Gain and Physical Activity in Pregnant Women With Obesity (Pas and Pes Study): Randomized Controlled Trial
Source: JMIR Mhealth Uhealth. 2022 Feb 15;10(2):e28886. doi: 10.2196/28886 (PMC8889480; doi:10.2196/28886)
Supplement: Multimedia Appendix 2 [file mhealth_v10i2e28886_app2.docx]

Appendix 2. Linear model of gestational weight gain variables with adjustment variables: age, BMI physical activity and study group

|  | | | **Gestational weight gain,** (kg/week) | |
| --- | --- | --- | --- | --- |
| **Adjustment variables** | | | ß | 95% CI |
| **Intercept** | |  | 0.85 | 0.09 to 1.62 |
| **Age,** (years) | |  | -0.001 | -0.01 to 0.008 |
| **BMI T0,** (kg/m^2^) | |  | -0.008 | -0.02 to 0.01 |
| **Physical activity** | | |  |  |
|  | Category I or low | | Reference | Reference |
|  | Category II or moderate | | -0.03 | -0.18 to 0.10 |
|  | Category III or high | | 0.008 | -0.15 to 0.17 |
| **Previous birth** | | |  |  |
|  | No | | Reference | Reference |
|  | Yes | | 0.057 | -0.16 to 0.04 |
| **Study group** | | |  |  |
|  | Control | | Reference | Reference |
|  | Intervention | | -0.14 | -0.25 to -0.03 |

ß= beta estimate; CI= confidence interval; BMI= body mass index; T= time.
