# Supplementary material for: Effectiveness of a Step Counter Smartband and Midwife Counseling Intervention on Gestational Weight Gain and Physical Activity in Pregnant Women With Obesity (Pas and Pes Study): Randomized Controlled Trial
Source: JMIR Mhealth Uhealth. 2022 Feb 15;10(2):e28886. doi: 10.2196/28886 (PMC8889480; doi:10.2196/28886)
Supplement: Multimedia Appendix 3 [file mhealth_v10i2e28886_app3.docx]

Appendix 3. Multinomial model of physical activity variable with adjustment variables: age, BMI physical activity and study group

|  | | | **Physical activity** | | |
| --- | --- | --- | --- | --- | --- |
| **Adjustment variables** | | | **Category II or moderate, OR (95% CI)** | | **Category III or high**  **OR (95% CI)** |
| **Age,** (years) | |  | | 1.04 (0.94- 1.16) | 0.97 (0.87- 1.10) |
| **BMI T0,** (kg/m^2^) | |  | | 1.04 (0.85- 1.28) | 0.91 (0.71- 1.18) |
| **Previous birth** | | |  | |  |
|  | No | | Reference | | Reference |
|  | Yes | | 0.97 (0.33- 2.84) | | 0.90 (0.26- 3.15) |
| **Study group** | | |  | |  |
|  | Control | | Reference | | Reference |
|  | Intervention | | 2.41 (0.79- 7.39) | | 3.90 (1.06- 14.34) |

OR=odd ratio; CI= confidence interval; BMI= body mass index; T= time.
